# 

**Published:** 1904-03-15

**Authors:** 


					﻿THE
DENTAL REGISTER
EDITED BY
NELVILLE S. HOFF, D. D.S.,
ANN ARBOR, MICH.
Vol.^tlR.	15, 1904.	No. 3.
PRICE, ONE DOLLAR A YEAR IN ADVANCE.
PUBLISHED MONTHLY BY
SAMUEL· A, CROCKER & CO.,
THE OHIO DENTAL AND SURGICAL DEPOT,
33 to 39 w. “Ill St.. Cineiiniiiti. O.
Entered at the Postoffice at Cincinnati, 0., as second-class mail matter.
				

